# The Association of Tree Pollen Concentration Peaks and Allergy Medication Sales in New York City: 2003–2008

**DOI:** 10.5402/2011/537194

**Published:** 2011-04-20

**Authors:** Perry E. Sheffield, Kate R. Weinberger, Kazuhiko Ito, Thomas D. Matte, Robert W. Mathes, Guy S. Robinson, Patrick L. Kinney

**Affiliations:** ^1^Departments of Pediatrics and Preventive Medicine, Mount Sinai School of Medicine, 1 Gustave L. Levy Pl., Box 1512, New York, NY 10029, USA; ^2^Department of Environmental Health Sciences, Mailman School of Public Health, Columbia University, New York, NY 10032-3727, USA; ^3^Department of Environmental Medicine, New York University School of Medicine, New York, NY 10016, USA; ^4^Urban Public Health Program, Hunter College, City University of New York School of Public Health, New York, NY 10010, USA; ^5^New York City Department of Health and Mental Hygiene, Bureau of Environmental Surveillance and Policy, New York, NY 10007, USA; ^6^Louis Calder Center, Biological Field Station, Fordham University, Armonk, New York, NY 10504-1104, USA

## Abstract

The impact of pollen exposure on population allergic illness is poorly characterized. We explore the association of tree pollen and over-the-counter daily allergy medication sales in the New York City metropolitan area. Dates of peak tree pollen (maple, oak, and birch) concentrations were identified from 2003 to 2008. Daily allergy medication sales reported to the city health department were analyzed as a function of the same-day and lagged tree pollen peak indicators, adjusting for season, year, temperature, and day of week. Significant associations were found between tree pollen peaks and allergy medication sales, with the strongest association at 2-day lag (excess sales of 28.7% (95% CI: 17.4–41.2) over the average sales during the study period). The cumulative effect over the 7-day period on and after the tree pollen peak dates was estimated to be 141.1% (95% CI: 79.4–224.1). In conclusion, tree pollen concentration peaks were followed by large increases in over-the-counter allergy medication sales.

## 1. Introduction

Increased concentrations of various pollens have been associated with higher rates of allergic sensitization [1, 2], tendency towards increased asthma episodes [3], higher numbers of asthma-related emergency department (ED) visits [4–7] and hospital admissions [8, 9], as well as higher numbers of allergic rhinitis ED visits [10] and physician visits [11]. Allergic rhinitis, a type of allergic airway disease that is a risk factor for increased asthma severity [12], decreases the quality of life of a substantial proportion of the United States population (10%–30% of adults and up to 40% of children) and imposes large costs on our health care system [13]. The direct costs for allergic rhinitis alone, including both health services and prescription medication sales, were $11.2 billion in 2005 [14]. Over-the-counter (OTC) allergy medication sales account for an increasing additional cost associated with allergic rhinitis [15, 16]. 

Allergy medication use is also believed to be associated with higher pollen concentrations although only one prior study was found looking specifically at this outcome [17], a French study that looked at prescription medications. In addition, there is variability in pollen types examined, demographics, temporal covariates, and health outcomes, with some studies suggesting interactions of pollen exposure with air pollution or personal characteristics [18]. Not all studies have shown positive associations between pollen and respiratory illness [19], and results have varied by pollen species [20]. Furthermore, lagged effects have not been consistently examined. Finally, there are no established systems for surveillance of pollen-related allergic illness over time. Thus, the temporal relationship between pollen peaks and fluctuations in morbidity due to allergic diseases is not well-characterized. 

Allergic airway disease primarily involves ambulatory care and symptomatic relief via self-administration of medications. Thus, studies that look at severe health outcomes such as ED visits and hospitalizations for respiratory illness only capture a small fraction of the population affected. However, records of more severe outcomes are more widely available and historically reliable. 

This study attempts to broaden the understanding of the association of pollen and population health behaviors by analyzing whether peaks in tree pollen concentration are associated with peaks in OTC allergy medication sales over a six-year period in New York City (NYC). By using a new method of minor illness surveillance, this data has the ability to capture an association of pollen with a more proximal indicator of allergic disease and thus possibly a larger fraction of the illness burden. 

## 2. Methods 

### 2.1. Data

#### 2.1.1. Pollen Data

Airborne pollen was collected with a Burkard volumetric spore trap (Burkard Manufacturing Co., Rickmansworth, UK)—Hirst-type sampler [21]**—**located on the rooftop at Fordham University's Louis Calder Biological Station in Armonk, NY about 30 miles north of Manhattan. This station is the closest long-term pollen record for the NYC region. Trained counters carried out microscopic analysis of pollen. Resulting daily pollen counts were converted into concentrations (particles per cubic meter of air) for the six years from 2003 to 2008. Peak dates for each pollen type in each year were identified. We focused on reviewing archival pollen slides from dates near expected seasonal peaks to identify the actual peak dates. We used peak pollen dates rather than daily pollen counts as the exposure metric, because peak dates are easily identified and are not affected by differential measurement methods and pollen counters. Thus, peak dates are more generalizable across different settings, and this analysis provides conservative estimates of the impact of pollen on allergy medication sales. Additionally, we were able to identify peaks without a completely continuous data set which will be the focus of future analyses. 

For this study, we analyzed daily concentrations of four genera of tree pollen: elm (*Ulmus* spp.), maple (*Acer* spp.), birch (*Betula* spp.), and oak (*Quercus* spp.). These subtypes were selected *a priori,* because they are clinically relevant aeroallergens in the U.S. [22] and have well-established sensitization patterns in populations from the northeast region of the U.S. [23, 24]. Other important pollens, including weed pollens such as ragweed and *Artemesia *sp. (sage/mugwort/wormwood) as well as grass pollens were excluded from the study in order to focus on earlyseason allergy. We defined “early-season” as March through May to capture the tree pollen peaks that are well known among allergists (per clinical pollen season charts) to occur in the northeastern U.S. during these months.

#### 2.1.2. Weather and Air Pollution Data

Daily meteorological data from LaGuardia International airport in New York was downloaded from the National Climatic Data Center. Daily average temperature was selected based on a review of previous literature. While preseason temperature and precipitation are positively correlated with tree pollen concentration [22], our study looks at short-term impact only. Data for particulate matter with aerodynamic diameters less than 2.5 microns (PM_2.5_) were obtained from U.S.EPA Air Quality System for NYC's five boroughs (21 sites). The temporal variations of PM_2.5_ across these monitors were highly correlated (*r* > 0.85). Therefore, we computed the average of multiple sites, taking into consideration the difference in site-specific means and standard deviations [25].

#### 2.1.3. Allergy Medication Sales Data

Data on OTC pharmacy sales are reported electronically to the NYC Department of Health and Mental Hygiene on a daily basis from over 200 store locations, disproportionately in Manhattan but also from the other four NYC boroughs (Table 1) and nearby suburbs in New York State and New Jersey. The store locations in this database cover approximately 30% of retail pharmacies in NYC [26]. 

 Although initially collected primarily for communicable disease surveillance, the data has served other purposes such as smoking cessation intervention evaluation [27]. For this analysis, the following brand-name and generic products were classified as allergy medications: Alavert, Benadryl, Claritin, loratidine, Sudafed, and Tavist, as well as other oral and nasal spray medications that include the word “allergy” in their name.

### 2.2. Data Analysis

Our main interest was the alignment (i.e., the timing and lag structure) of the tree pollen peaks and major peaks in the allergy medication sales. Since the absolute magnitude of the pollen peaks can vary geographically within NYC, and because we used pollen data from a single station outside the city, absolute peak values from this database may not accurately reflect the pollen exposures of the entire city's residents. Therefore, we used an indicator variable (1 for peak dates; 0 otherwise) for the tree pollen peak dates, rather than the absolute pollen concentration. We defined “tree pollen peak date” to be the date with the highest pollen concentration from available data during the March through May period each year for each genus. There were two peak dates for elm in 2007 and 2008 because there were two dates with the same pollen concentration in each of those years. For the other species, there was only one peak date in each year. Thus, we had a total of 26 pollen peak dates over the six-year study period. However, 18 tree pollen peak dates were included in the analysis because elm was excluded due to no corresponding major short-term peaks in allergy medication sales.

We developed a regression model to estimate the impact of the 18 tree pollen peak dates on the daily allergy medication sales, adjusting for potential confounding factors. Since we expected delayed effects and multiday effects, we examined lags 0 through 6 days from the pollen peak dates (i.e., we compared today's allergy medication sales with today's tree pollen peak, with yesterday's pollen peak, etc.). To test consistency with a causal relationship, we also examined the “wrong side” of the lag up to 6 days (i.e., we compared today's allergy medication sales with tomorrow's pollen peak, with the day after tomorrow's pollen peak, etc.). We first included individual lags of the tree pollen peak date indicator to determine the lag structure of associations, and then included all seven day lags to estimate the multiday effects (i.e., unconstrained distributed lag model).

We included the following five temporal covariates in the regression model: a day-of-week indicator variable as a factor variable to fit the weekly sales pattern (i.e., fewer sales on weekends), a year indicator variable to account for year-to-year variability in the medication sales, and three temperature variables to capture the effects of temperature on allergy symptoms [28] or on purchasing behaviors. Since we do not know the functional form of the relationship, we fitted a smooth function of temperature using natural cubic splines with three degrees of freedom to allow a U-shaped relationship. Likewise, we included the average of 1- through 3- day lagged temperature to model the delayed effect, with natural cubic splines with three degrees of freedom. Since there are general upward and downward trends in allergy medication sales within the March through May period that may not be associated with pollen, we included a smooth function of study days using natural cubic splines with 8 degrees of freedom within each season. We chose 8 degrees of freedom based on penalized splines that determine the optimum effective degrees of freedom [29] given no explanatory variables to force the shape of the trend. However, since the seasonal trend in allergy medication sales within our study period may also reflect the actual effect of pollen, our main model conservatively estimates the impact of pollen. Therefore, we conducted sensitivity analysis using natural cubic splines with 6, 4, and 2 degrees of freedom. We conducted further sensitivity analysis on models without adjustment for seasonal trend, year-to-year variation, and temperature. Finally, pollen peak dates may coincide with days when air is stagnant, and therefore air pollution is high. In order to address possible confounding, we also analyzed the city-wide average fine particle (PM_2.5_) concentration at lag 0 through 6 days using the same main model we used to estimate the impact of pollen. The effect of pollen was also estimated with PM_2.5_ in the model. 

To assess potential geographic differences in the impact of pollen on allergy medication sales, we also applied the main model described above separately to each of the five boroughs and the area outside of NYC. 

Daily allergy medication sales exhibited a highly skewed distribution. We, therefore, conducted the analysis described above with log-transformed outcome data. The extent of the impact was expressed as percent excess from the average allergy medication sales as measured by number of units sold during March through May during the study years, adjusting for the covariates described above. Analysis was conducted using statistical software R version 2.10 (R Core Development Team, 2009).

## 3. Results

**Figure 1:**
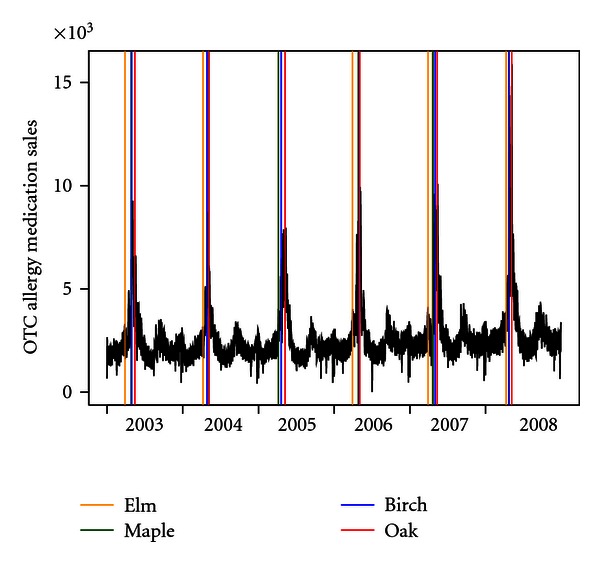
Time-series plot of daily allergy medication sales. Superimposed lines are dates of tree pollen peaks color coded by genera.

Figure 1 shows time-series plots of OTC allergy medication sales for the entire city from 2003 to 2008, with tree pollen peak dates superimposed. While there is a general upward trend in allergy medication sales each spring before the pollen peak dates, the pollen peak dates do appear to coincide with sharp peaks in the medication sales. The increase in sales can partially be explained by the changes in number of stores reporting, which increased from 206 in 2003 to 231 in 2008. Table 1 shows the data distribution of allergy medication sales by borough, temperature, and PM_2.5_ concentration. The temporal pattern of allergy medication sales were highly correlated across boroughs. 

**Figure 2:**
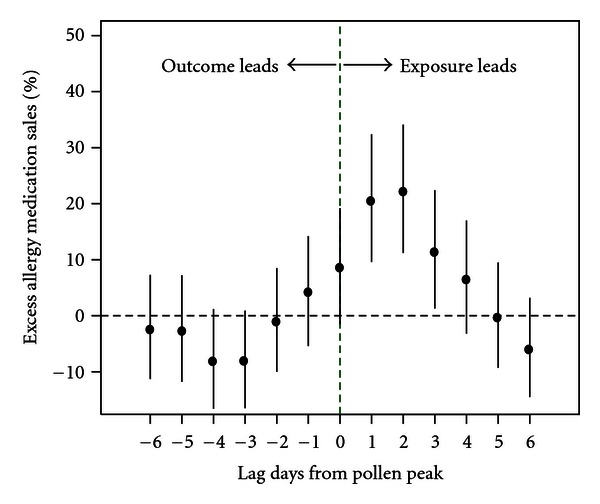
Estimated impacts of the tree pollen peaks on the percentage change in the mean allergy medication sales during March through May of the study period. Each individual lagged peak indicator variable was included separately in the model.

Figure 2 shows the estimated impacts of tree pollen peaks on percentage change in allergy medication sales at specific lags. Significant associations were found at lags 1 through 3 days. No significant association was found on the “wrong side” of lagged associations (i.e., with allergy medication sales leading the pollen exposure). 

**Figure 3:**
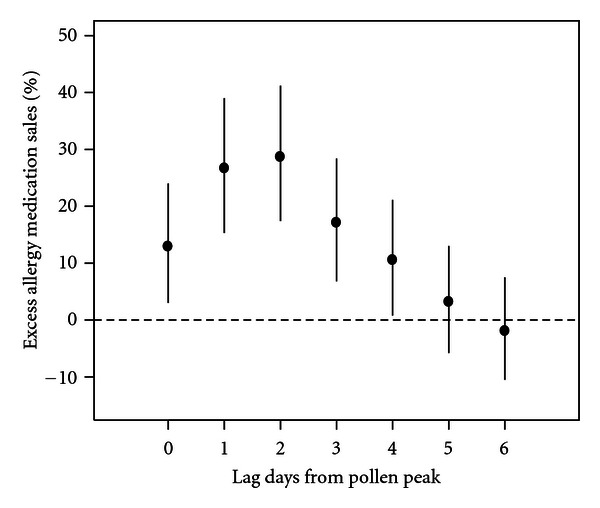
Estimated impacts of the tree pollen peaks on the percentage change in the mean allergy medication sales during March through May during the study period. All the lagged peak indicators were included in the model.

**Figure 4:**
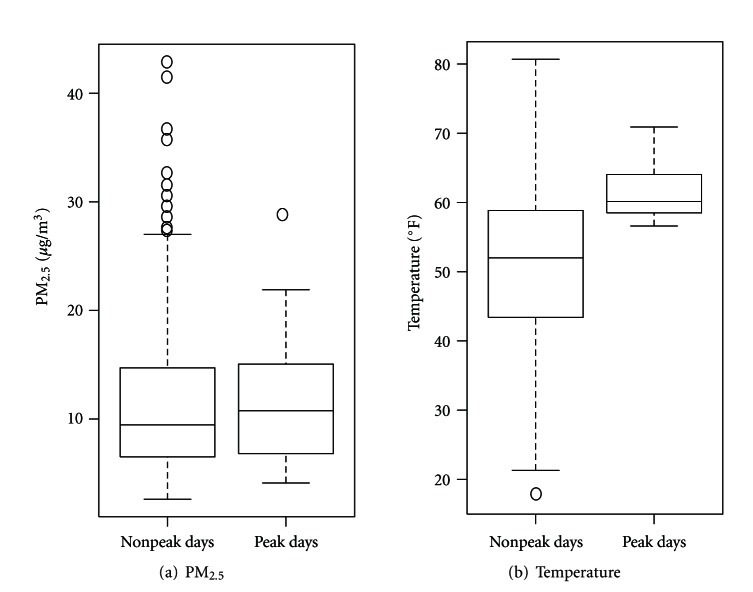
PM_2.5_ levels and temperature by nonpeak days versus peak days of pollen.

Figure 3 shows the estimated impacts of tree pollen peaks when all lagged peak date indicators were included simultaneously. The largest statistically significant impact occurred at 2-day lag (28.7% [95% CI: 17.4, 41.2]), followed by lag 1 day. In the distributed lag model, the sum of the effects over the seven-day period was 141.1% (95% CI: 79.4, 224.1). Table 2 shows numerical results for all the lagged pollen peak indicator variables from our main model (shown in Figure 3) which is fully adjusted, as well as results sorted by subarea, results from sensitivity analysis, and estimated percent excess risk for PM_2.5_. The estimated pollen impacts were highest in the Bronx and lowest in Staten Island but were generally comparable across boroughs. Adjusting for PM_2.5 _ slightly decreased risk estimates for pollen, but PM_2.5_ itself was not associated with allergy medication sales at any of the lags examined (see the last row of Table 2). PM_2.5_ levels were comparable between pollen-peak days and non-peak days (Figure 4). Not adjusting for year to year variation only slightly changed the pollen impact estimates. Not adjusting for temperature moderately increased the estimated pollen impact. This finding may in part be explained by the fact that pollen peak dates tend to occur in the warmer part of the March through May period (Figure 4). Removal of the seasonal trend term or less aggressive adjustment for the seasonal trend (i.e., using 2 or 4 degrees of freedom) substantially increased the pollen impact estimates. Most importantly, these alternative models showed essentially the same lag structure of associations.

## 4. Discussion

We found that OTC allergy medication sales increased significantly on the day of a pollen peak for maple, oak or birch, and that the increase persisted for 3 days after the peak. The maximum single-day increase in sales—~29%—occurred 2 days after a tree pollen peak. The multiday effect over one week was estimated to be 141%. Our findings indicate that monitoring OTC medication sales may be a useful method of population surveillance for allergic illness and the impact of pollen, as well as a potential measure of consumer anticipation of pollen events in response to public health advisories or other messaging. 

Our findings are generally consistent with other studies examining the relation of ambient pollen to minor allergic illness. In an urban area in France, a study found that daily purchases of prescription allergy medications were associated with same-day concentrations of grass pollen and some tree pollens while controlling for weather and air pollution [17]. A study in Ottawa, Canada, found no effect of tree pollen on ED visits for conjunctivitis and rhinitis, but ragweed and fungal spore concentrations appeared to be associated with same day ED visits while controlling for weather and air pollution. The exploration of lagged effects was not described in detail by the authors [10]. In Toronto, Canada, physician visits among the elderly for allergic rhinitis were associated with 10-day average ragweed concentrations but not with air pollution [11]. One strength of our study is that it includes a fuller examination of lags than these previous studies. 

The estimated effects of pollen on allergy medication sales were sensitive to adjustment for the seasonal trend within the March through May period. Using 8 degrees of freedom to fit the broad trend in the allergy medication sales may overcontrol for season, therefore underestimating the impact of tree pollen. Broad increases in medication sales occurred around the dates of pollen peaks. Much of the broad increase is likely explained by our seasonal covariate rather than our intermittent pollen peak indicator. Another limitation of our model is that it is possible that some individuals respond to pollen with a longer delay than seven days. It is also likely that pollen concentrations lower than the peak day concentration also cause allergic symptoms that contribute to medication sales. Furthermore, the model does not account for the impacts of re-entrainment/resuspension of pollen particles and ongoing pollen production from other sources that may persist for days without producing distinct peaks and yet affecting the population. This study focused on determining the temporal relationship between the sharp peaks in tree pollen and allergy medication sales rather the total impact of pollen over the season. 

As far as we are aware, our study is the first to use OTC allergy medication sales directly reported from pharmacies as an indicator of allergic illness. An advantage of this approach is that this health data reflects minor illness, as many will not seek health care nor have claims filed for prescription medications. The observed associations support use of genus-specific tree pollen season charts in clinical allergy practice, which are not currently being used in allergy clinics in New York (personal correspondence, President of the NY Allergy Society, January 2010). 

Limitations of this approach include the possibility that individuals may self-medicate using previously purchased OTC medications, that the single purchase of OTC allergy medication could result in usage at multiple times other than the day of purchase, and that available in-home medications may vary within a calendar year. Thus, our analysis likely underestimates the overall contribution of pollen to use of OTC allergy medications. Furthermore, purchase of an OTC allergy medication does not describe frequency of use, severity of symptoms, nor the number of individuals using a particular medication. Additionally, no demographic data about those purchasing the allergy medications is available. Conclusions about the observed increase in total allergy medication sales over the study period representing an increase in morbidity might be premature as the trend could also be the result of medications formerly requiring a prescription being sold as OTC products and therefore reflect the public response to increased advertising of these products. 

Work is needed on the specific magnitude of health impacts of aeroallergens, such as attributable burden of disease not only to allergic rhinitis but also to asthma, subpopulation sensitivity, better understanding of pollen type sensitivities, and rate of progression of pollen-induced symptoms to more severe health events. In addition, efforts to better detail the role of weather and climate variables as effect modifiers in pollen-related health impacts, the geospatial variation of pollen concentrations, and the timing and intensity of the pollen season will contribute to work in this field. Future work should explore relationships between meteorologic, variables and pollen using continuous daily concentration data and not just peak measures. 

## 5. Conclusions

Findings from this study suggest that peaks in maple, oak, and birch pollen concentrations may have prognostic value in anticipating acute allergic responses in the study region. Early warning of spring pollen peaks based on ongoing surveillance and meteorological forecast models could potentially inform clinical practice and reduce morbidity. The importance of this work lies not only in its potential contribution to public health surveillance and clinical guidance but also in its ability to inform projections of future pollen-related morbidity under a changing climate.

## Figures and Tables

**Table 1:** Distribution of daily allergy medication sales, temperature, and fine particles (PM_2.5_), March–May, 2003–2008.

Percentile	5th	25th	50th	75th	95th
Allergy medication sales (units)					
All boroughs	1792	2594	3302	4903	7984
Manhattan	1151	1798	2314	3383	5554
Bronx	51	66	86	130	270
Brooklyn	176	229	294	435	814
Queens	184	230	308	464	844
Staten Island	36	51	68	88	138
Outside NYC	125	159	208	300	491

Environmental variables					
Temperature (Degrees F)	34	44	53	59	70
Fine particles (ug/m^3^)	4	6	10	15	25

**Table 2:** Estimated percent excess allergy medication sales at lags 0 through 6 days from pollen peak dates and PM_2.5_ (last row).

Models	Lag 0	Lag 1	Lag 2	Lag 3	Lag 4	Lag 5	Lag 6
Main model (all boroughs)	13.0 (3.0, 24.0)	26.6 (15.3, 39.0)	28.7 (17.4, 41.2)	17.1 (6.8, 28.4)	10.5 (0.8, 21.1)	3.2 (−5.8, 13.0)	−1.9 (−10.5, 7.5)
By location of sales							
Manhattan	13.5 (3.4, 24.6)	26.7 (15.3, 39.2)	27.7 (16.4, 40.1)	17.4 (6.9, 28.8)	11.4 (1.6, 22.1)	3.8 (−5.3, 13.8)	−1.2 (−9.9, 8.3)
Bronx	15.1 (0.9, 31.2)	29.7 (13.6, 48.0)	38.3 (21.3, 57.6)	19.9 (5.2, 36.7)	10.9 (−2.6, 26.3)	0.7 (−11.5, 14.6)	−2.3 (−14.1, 11.2)
Brooklyn	11.4 (0.3, 23.8)	27.9 (15.1, 42.1)	27.9 (15.3, 42.0)	15.5 (4.0, 28.3)	6.8 (−3.7, 18.4)	0.7 (−9.2, 11.7)	−5.3 (−14.6, 5.0)
Queens	14.6 (3.1, 27.3)	29.1 (16.1, 43.5)	29.8 (16.9, 44.1)	16.5 (4.8, 29.4)	7.7 (−2.9, 19.5)	2.1 (−8.0, 13.2)	−1.1 (−10.8, 9.7)
Staten Island	5.3 (−6.0, 18.1)	14.8 (2.4, 28.8)	16.4 (3.9, 30.3)	7.1 (−4.4, 20.0)	12.2 (0.2, 25.5)	−1.1 (−11.6, 10.6)	−1.3 (−11.7, 10.4)
Outside NYC	10.4 (−0.2, 22.2)	24.8 (12.7, 38.2)	31.7 (19.1, 45.7)	16.4 (5.2, 28.8)	9.4 (−1.0, 20.9)	3.2 (−6.6, 14.1)	−3.8 (−13.0, 6.3)

Sensitivity analysis (all boroughs)							
With PM_2.5_	9.5 (−0.6, 20.7)	23.0 (11.8, 35.3)	24.6 (13.4, 37.0)	13.8 (3.4, 25.1)	7.4 (−2.4, 18.2)	0.9 (−8.2, 11.0)	−3.0 (−11.8, 6.6)
Without year indicator	12.5 (0.9, 25.5)	25.9 (12.8, 40.5)	28.1 (15.0, 42.8)	16.7 (4.6, 30.1)	10.1 (−1.1, 22.7)	2.6 (−7.9, 14.2)	−2.5 (−12.5, 8.5)
Without temperature adjustment	20.6 (9.4, 32.8)	35.8 (23.2, 49.6)	35.0 (22.6, 48.7)	20.5 (9.4, 32.8)	13.0 (2.6, 24.5)	4.6 (−5.1, 15.2)	−1.0 (−10.2, 9.0)
Seasonal trend with d.f. = 6	12.4 (2.4, 23.3)	26.1 (14.8, 38.4)	28.6 (17.2, 41.0)	17.4 (7.0, 28.8)	11.0 (1.3, 21.7)	3.8 (−5.2, 13.8)	−1.1 (−9.8, 8.3)
Seasonal trend with d.f. = 4	16.3 (5.9, 27.8)	30.3 (18.6, 43.2)	32.3 (20.5, 45.3)	20.3 (9.5, 32.2)	13.4 (3.3, 24.5)	5.8 (−3.6, 16.1)	0.4 (−8.5, 10.2)
Seasonal trend with d.f. = 2	31.1 (17.4, 46.5)	49.0 (33.4, 66.6)	50.5 (34.8, 68.0)	37.5 (23.1, 53.6)	29.8 (16.4, 44.9)	23.6 (10.8, 37.8)	17.4 (5.3, 30.8)
Without seasonal trend	36.7 (21.5, 53.9)	54.8 (37.5, 74.2)	55.5 (38.3, 74.9)	41.8 (26.0, 59.6)	33.6 (18.8, 50.3)	27.7 (13.6, 43.6)	21.0 (7.7, 36.1)

PM_2.5_ Effects per 10 ug/m^3^	−1.9 (−4.8, 1.2)	−3.2 (−6.4, 0.1)	−0.7 (−4.0, 2.7)	−1.2 (−4.4, 2.2)	−0.8 (−3.9, 2.5)	−1.2 (−4.2, 2.0)	0.2 (−2.5, 3.0)
